# Tendency to call an ambulance or attend an emergency department for minor or non-urgent problems: a vignette-based population survey in Britain

**DOI:** 10.1136/emermed-2020-210271

**Published:** 2022-03-10

**Authors:** Alicia O'Cathain, Rebecca Simpson, Miranda Phillips, Emma Knowles

**Affiliations:** 1 School of Health and Related Research, The University of Sheffield, Sheffield, UK; 2 NatCen Social Research, London, UK

**Keywords:** pre-hospital care, utilisation, health service accessibility, urgent care

## Abstract

**Background:**

There are concerns about high levels of demand for emergency health services. The aim was to identify the characteristics of the British population with a tendency to contact emergency medical services and EDs for minor or non-urgent problems.

**Methods:**

A survey of the British adult population in 2018. Six vignettes were constructed about illness in adults (cough/sore throat or diarrhoea/vomiting), injury in adults (sore rib or back pain) and fever in children (occurring weekday or weekend).

**Results:**

The response rate was 42%, with 2906 respondents. 11% (319/2716) of respondents selected to contact an ambulance and 43% ED, mainly for the vignettes about fever in children and sore rib. Males, people from ethnic minority communities and older people had a tendency to contact emergency services for minor problems. Tendency to call an ambulance was also characterised by ‘low resources’ (manual or unskilled occupations, no car, low health literacy), worry that a symptom might be serious, distress (feeling overwhelmed by health problems) and frequent use of EDs. For EDs, there was an attraction to EDs because of availability of tests.

**Conclusion:**

Whereas use of emergency ambulances for minor or non-urgent problems appeared to be driven by people’s lack of resources, including lack of transport, use of EDs appeared to be driven by their attractive characteristic of offering tests quickly.

**Key messages**

**What is already known on this subject**

- Research, including systematic reviews, has identified factors affecting demand for emergency ambulances and EDs for minor or non-urgent problems.
- Studies have tended to focus on service users of either emergency ambulances or EDs.

**What this study adds**

- We undertook a population survey using vignettes of minor or non-urgent conditions to identify the factors affecting tendency to contact emergency ambulances or EDs.
- The tendency to call an ambulance was characterised by ‘low resources’ (manual or unskilled occupations, low health literacy, lack of car) and distress (feeling overwhelmed when faced with health problems).
- The tendency to contact an ED was characterised by an attraction to EDs in terms of availability of tests.

## Introduction

There is concern about increasing demand for emergency medical services and EDs. For example, demand for emergency medical services increased by 1.4% per annum in Australia, with the rate of increase considerably higher for transport of patients not requiring medical intervention from paramedics.^1^ Some researchers have focused on understanding the demand for what are called minor, non-urgent, non-serious or low-acuity health problems, in order to reduce the demand for emergency care overall. Estimates of the incidence of this type of demand vary internationally from 8% to 62%, with an average of 37%.^2^

A considerable amount of research has been undertaken on this topic, including systematic reviews to synthesise the evidence. Four recent reviews summarise the evidence well: a systematic review of use of emergency ambulances for primary care-sensitive conditions,^3^ two systematic reviews of non-urgent or self-referral to EDs^2 4^ and a rapid review of why patients choose to access a range of emergency and urgent care services.^5^ Only one of these reviews considered quantitative studies of sociodemographic characteristics of patients.^2^ This review focused on non-urgent use of EDs and found that younger adults, Black people and people on low income were more likely to have non-urgent visits, although a number of studies showed no association with these characteristics. Findings about gender were inconclusive.^2^ Although not measured quantitatively, socioeconomic issues of deprivation and having no transport were identified in another of the reviews.^3^ Patient-related factors affecting this type of demand included: poor physical health and comorbidities,^2 3^ concerns about health,^2 4^ personal anxiety and risk management,^3^ health knowledge^3^ and patients taking advice from family or health professionals to call an ambulance or attend an ED.^2–5^ Service-related factors included perceived poor access to primary care,^2–5^ patients having no confidence in primary care or alternative services,^4^ perceived need for treatment and investigations available at EDs,^4 5^ the convenience of EDs^2 4 5^ and the cost of attending alternative services to EDs.^2 4 5^

Studies have tended to be service specific, and systematic reviews have tended to be based on a mixture of service users and health professionals’ perspectives of care seeking.^3–5^ We wanted to understand people’s help-seeking behaviour from their perspectives rather than health professionals’ perspectives, and from the perspective of an emergency and urgent care system as recommended by a recent review.^5^ So we undertook a study focusing on demand for emergency and urgent care for non-urgent or minor problems from the perspective of patients and the public. First, we undertook a realist review to identify programme theories or explanations for help-seeking behaviour based on qualitative interviews with patients.^6^ We found some explanations that aligned with findings from previous systematic reviews: anxiety caused by uncertainty of symptoms; following advice from trusted others; perceptions that they would receive a better service from emergency care than other services; and frustration at a lack of accessibility of primary care. We also found other explanations not addressed in these systematic reviews: anxiety caused by past traumatic events; the need to get back to normal quickly; the need for immediate pain relief; and inability to cope due to stressful lives.^6^

We aimed to undertake a population survey to test the effect of a variety of sociodemographic, patient-related and service-related factors identified in previous research on population tendency to use emergency services for minor or non-urgent problems.

## Methods

### Design

We undertook a cross-sectional survey of the British population in 2018, including vignettes of actions they would take when faced with minor or non-urgent problems.

### Patient and public involvement

We took the idea for the wider study to the Sheffield Emergency Care Forum, a patient and public involvement (PPI) group dedicated to emergency care research.^7^ We discussed the study and two members of the forum joined our funding application as coapplicants. Our two PPI coapplicants and other members of Sheffield Emergency Care Forum attended project management groups where results were presented and discussed, influencing interpretation and further analysis. When developing the questionnaire for the survey reported here, we held a workshop with 13 members of the public including our PPI coapplicants where potential questions were presented and discussed.

### Sampling

NatCen Social Research conducts a survey called the British Social Attitudes Survey each year. It measures the social attitudes of a representative sample of British adults aged 18 years and over.^8^ In 2018, NatCen selected 395 of 9000 postcode sectors in Britain with probability proportional to the number of addresses in each sector. Then they selected 26 addresses in each sector. This produced 10 270 addresses for interviewers to visit and list those aged 18 or over before randomly selecting one adult for interview. People living in institutions were not included. The selected sample was divided into four parts. Each part consisted of approximately 1000 respondents and each part was nationally representative. We used funding from the National Institute for Health Research to ask a set of questions to three parts of the sample. We chose this sample size of 3000 because it offered statistical power for subgroup analyses.

### Mode of administration

NatCen used face-to-face computer-assisted interviews to administer most questions on the questionnaire. They sent a letter to selected addresses to say that an interviewer would visit, and included an unconditional incentive of a gift voucher. Interviewers attended the addresses and administered the questionnaire face to face. Some of the questions were asked as a self-completed paper questionnaire. Interviewers handed this self-completed part of the questionnaire to respondents at the end of interview. This part of the questionnaire was collected later by the interviewer or posted to NatCen. Some people who had completed the interviewer-administered part of the questionnaire did not return the self-completed part so the response rate to this part was lower. The survey occurred in July to November 2018.

### The content of the questionnaire

Our questions were embedded within a wider British Social Attitudes questionnaire which covered a range of topics. Our questions focused on help seeking for what we termed ‘unexpected health problems that were not life threatening’ because people do not necessarily understand the meaning of the words ‘urgent’ and ‘emergency’.^9^

We aimed to measure the sociodemographic, patient-related and service-related factors within the questionnaire. Sociodemographic factors were collected by the British Social Attitudes Survey every year. We selected 10 of these variables to test in our analysis. For patient-related and service-related factors, we listed factors from previous research and then constructed questions to measure these factors. These questions were discussed at a large PPI meeting (see earlier section) where members of the public attempted to complete them and then fed back problems to the team.

To measure the tendency to contact services, three researchers (the lead investigator of the study, an ED consultant and a general practitioner) constructed three pairs of vignettes within the questionnaire. Each survey respondent responded to three vignettes, one from each pair (table 1). The symptoms in all of these vignettes were considered as too minor or non-urgent to call an ambulance or attend an ED by both the two clinicians and a national resource for patients (https://www.nhs.uk/), which describes self-care as usually adequate for these symptoms. The options offered to respondents for each vignette were presented by level of urgency, with an emergency ambulance as the most urgent: call 999 for an ambulance, go to the A&E department, contact a General Practitioner (GP) (for telephone advice or an appointment—including GP ‘out of hours’ service), go to another NHS service, for example, walk-in centre, minor injury unit, pharmacy/chemist, call 111, deal with the problem myself, none of these. Respondents could select more than one option within the same vignette. The two ‘child illness’ vignettes deliberately had different times at which the problem occurred to address decision-making when different services were available.

**Table 1:** Vignettes used in the questionnaire

Type of vignette	Pair 1	Pair 2
Illness	Imagine you have had a cough and sore throat for 3 days.	Imagine you have had diarrhoea and vomiting for 2 days.
Injury	Imagine you have fallen and have a very painful rib and it is 20:30 in the evening.	Imagine you have had back pain for 2 weeks and have not been able to sleep.
Child	Imagine your young child or a young child in your care has a high temperature and cried throughout yesterday and last night. Today, which is a Saturday, you do not think the child has improved.	Imagine your young child or a young child in your care has a high temperature and cried throughout yesterday and last night. Today, which is a Wednesday, you do not think the child has improved.

The questionnaire was then piloted twice by NatCen on 50 members of the public, with improvements made to questions after each pilot.

### Analysis

We undertook two analyses. First, we compared the respondents selecting emergency ambulance as their highest urgency option for any of the vignettes and compared them with respondents selecting other services or self-care as one of their highest urgency options. Second, we removed the respondents selecting ambulance as their highest urgency option and compared those selecting ED as their highest urgency option with the remaining respondents. To do this, we created two binary outcome variables. One was based on whether respondents chose to contact an emergency ambulance versus less urgent options. The other was based on whether respondents chose to attend an ED versus less urgent options. Options of refusal/don’t know/missing and ‘none of the above’ were excluded from the analysis.

In our original analysis we tested 53 factors which we grouped into three sets for presentation purposes only; all factors were tested together. Each factor is described in detail elsewhere.^10^ There were 10 sociodemographic items, 15 patient-related items and 28 service-related items. Health knowledge was in the patient-related factors and was measured using the Health Literacy Questionnaire domains of ‘understanding information’ and ‘ability to communicate with health professionals’.^11^ We undertook logistic regression using SPSS V.25.^12^ First, we undertook a univariate analysis, testing each of the 53 independent variables. We did this because we intended to use stepwise backward elimination and the model would not run using all 53 variables. Then we tested the statistically significant variables (where p<0.05) in a complete case multivariable logistic regression. We used stepwise backward elimination with a cut-off of 0.05 for selection. We treated non-response to survey questions as missing data. Finally, we tested for differences between ‘vignette set’ by testing interaction terms between ‘vignette set’ completed and each of the factors in the final multivariable models.

A statistical reviewer raised concerns about this analysis and recommended that we test a smaller number of highly relevant variables and fit all of those variables in a single multivariable analysis to reduce concerns about spurious exclusion of variables in a stepwise backward elimination approach. So we limited the set of 53 variables to 25 by only including variables identified as affecting service use in the systematic reviews described earlier in this paper,^2–6^ using only one variable to address each issue from the systematic reviews (our original analysis used two to three variables to address some issues). There was uncertainty about the effect of some of these variables in the systematic reviews^2–5^ and one review identified variables that had not been tested quantitatively so our study built on these systematic reviews.^6^ We then tested each variable in a univariate analysis for ambulances and then for EDs. We selected the variables that were statistically significant for either service (p<0.05). These 20 variables were tested in a multivariable analysis for ambulance and for EDs separately. We report this analysis here and then compare the results with our original approach. We present the results as ORs with 95% CIs.

## Results

### Response rate and non-response bias

The full survey had a response rate of 42%. A total of 2906 respondents completed the items in the face-to-face part of the questionnaire. Response rates were higher where there was easy physical access to a property or the property was in better condition than those around it.^8^ A total of 2309 out of 2906 (79%) of those completing the face-to-face part of the questionnaire also returned the self-completed part.

### Description of sample

The sample has been described in detail elsewhere.^10^ Fourteen per cent of the sample were 75 years old and over, 11% were from ethnic minority communities and 23% reported fair or poor health.

### Tendency to contact emergency services

Responses to the vignettes are shown in table 2. Eleven per cent (319/2716) of respondents selected the option to call an ambulance, mainly for the scenarios involving childhood fever and a sore rib (table 2). Forty-three per cent (1175/2716) selected to attend an ED, again mainly for childhood fever and sore rib. When those selecting ambulance as their most urgent option were removed, 1032 respondents remained for whom ED was the most urgent option selected. The percentages with a tendency to contact an ambulance were very similar for each child vignette. The percentages with a tendency to contact an ED were also very similar for each child vignette.

**Table 2:** Percentages of population selecting options for different vignettes*†

	Adult illness		Adult injury		Child illness		Any
	Cough n (%)	Diarrhoea and vomiting n (%)	Sore rib n (%)	Back pain n (%)	Saturday n (%)	Wednesday n (%)	n (%)
Call 999 for an ambulance.	4 (0.3)	21 (1.5)	76 (5.7)	13 (0.9)	126 (9.4)	117 (8.5)	319 (11.7)
Go to the A&E department.	17 (1.3)	70 (5.1)	357 (26.6)	88 (6.4)	476 (35.4)	439 (32.0)	1175 (43.3)
Contact a GP (for telephone advice or an appointment—including GP ‘out of hours’ service).	306 (22.8)	626 (45.6)	171 (12.7)	986 (71.9)	378 (28.1)	696 (50.7)	1883 (69.3)
Go to another NHS service, for example, walk-in centre, minor injury unit, pharmacy/chemist.	249 (18.5)	255 (18.6)	271 (20.2)	245 (17.9)	336 (25.0)	263 (19.2)	1178 (43.4)
Call 111.	40 (3.0)	219 (16.0)	316 (23.5)	91 (6.6)	408 (30.4)	298 (21.7)	1037 (38.2)
Deal with the problem myself.	972 (72.3)	673 (49.1)	365 (27.2)	271 (19.8)	43 (3.2)	43 (3.1)	1794 (66.1)
n=100% of respondents	1344	1372	1344	1372	1344	1372	2716

*Distribution of responses with emergency ambulance included.

†Multiple options could be selected for each vignette so the percentages add to more than 100.

For the univariate analysis comparing the characteristics of those who would or would not choose an emergency ambulance, we compared the 319 who chose an ambulance in any vignette with the 2397 who selected only lower urgency options (n=2716). For the ED analysis, we compared the 1032 whose most urgent option was the ED to the 1347 who chose only lower urgency options (n=2379). For the multivariable complete case analysis, 2062 were included in the emergency ambulance analysis and 1922 in the ED analysis.

### Sociodemographic factors associated with tendency to contact emergency services

Seven sociodemographic factors were tested in the univariate analyses. All were statistically significant for either ambulance or ED and were included in multivariable analyses (table 3). Tendency to contact both emergency ambulances and EDs was higher for older people, men and people from ethnic minority communities in the multivariable analyses (table 3). For ambulance services, there were a further two variables, both related to lack of resources: people from manual and unskilled occupations, and people without cars (as a proxy for lack of transport).

**Table 3:** Sociodemographic factors associated with tendency to contact an emergency service

Factors	Ambulance versus less urgent options n=2716		ED versus less urgent options n=2397	
	Multivariable model OR (95% CI)	n	Multivariable model OR (95% CI)	n
Age		P=0.001		P<0.001
18–24	1	94	1	87
25–34	1.7 (0.7 to 4.5)	268	0.9 (0.5 to 1.5)	241
35–44	1.2 (0.5 to 3.3)	307	0.9 (0.6 to 1.6)	286
45–54	1.3 (0.5 to 3.5)	351	1.4 (0.8 to 2.3)	327
55–64	1.7 (0.7 to 4.4)	384	1.6 (0.9 to 2.5)	345
65–74	**3.3 (1.3 to 8.3)**	396	**1.8 (1.1 to 3.0)**	337
75+	**3.0 (1.2 to 7.6)**	262	1.1 (0.6 to 1.9)	211
Sex		P=0.004		P=0.006
Female	1	1185	1	1077
Male	**1.4 (1.0 to 1.9)**	877	**1.3 (1.1 to 1.7)**	457
Ethnicity		P=0.015		P=0.010
White	1	1894	1	1700
Ethnic minority	**1.8 (1.1 to 3.0)**	168	**1.7 (1.1 to 2.5)**	134
Social class based on occupation		P=0.007		P=0.407
I Professional	1	152	1	145
II Managerial	1.6 (0.7 to 3.8)	795	1.0 (0.7 to 1.5)	732
IIIn Skilled non-manual	1.4 (0.6 to 3.4)	423	1.1 (0.7 to 1.6)	388
IIIm Skilled manual	**3.2 (1.3 to 7.7)**	298	1.3 (0.8 to 2.1)	239
IV and V Partly skilled and unskilled	**2.4 (1.0 to 5.7)**	366	1.3 (0.9 to 2.1)	305
Armed forces	1.7 (0.4 to 7.9)	28	1.0 (0.4 to 2.5)	25
Deprivation		P=0.347		P=0.682
5 Affluent	1	456	1	413
4	0.7 (0.4 to 1.2)	481	1.1 (0.8 to 1.6)	440
3	0.8 (0.5 to 1.3)	403	1.1 (0.8 to 1.5)	367
2	1.1 (0.7 to 1.7)	352	0.9 (0.7 to 1.2)	301
1 Deprived	0.7 (0.4 to 1.2)	370	0.9 (0.7 to 1.2)	313
Rural/Urban		P=0.663		P=0.901
Urban	1	1558	1	1377
Rural	0.9 (0.6 to 1.4)	504	1.0 (0.8 to 1.3)	457
Car ownership		P<0.001		P=0.811
1 or more cars	1	1103	1	1023
No car	**2.5 (1.6 to 3.8)**	254	1.0 (0.7 to 1.3)	198
Not included†	**1.9 (1.3 to 2.7)**	705	0.9 (0.8 to 1.2)	613

† this question on car ownership was not included in all the questionnaires

emboldened numbers are where 95% CI does not include 1

### Patient-related factors associated with tendency to contact emergency services

Eleven patient-related factors were tested in the univariate analyses for ambulance and ED. Eight were statistically significant for either ambulance or ED. These eight were included in the multivariable analyses (table 4). In the multivariable analysis, tendency to call an emergency ambulance in these non-urgent scenarios was higher for people with lower health literacy, those who were anxious about symptoms such as pain and those who felt overwhelmed when they had an unexpected health problem (table 4). A factor in the ambulance multivariable analysis was difficult to interpret. People were asked how likely they were to seek advice from family and friends if faced with this scenario. Those who were fairly likely to seek advice were less likely to tick that they would call an emergency ambulance. This relationship was not seen for those who were very likely to seek advice from family and friends, making it difficult to interpret.

**Table 4:** Patient-related factors associated with tendency to contact emergency services in the non-urgent scenarios

Factors	Ambulance versus all other options		ED versus all other options, excluding ambulance	
	Multivariable model		Multivariable model	
	OR (95% CI)	n	OR (95% CI)	n
**Health**				
General health		P=0.122		P=0.294
Excellent	1	197	1	180
Very good	0.8 (0.4 to 1.5)	661	1.0 (0.7 to 1.5)	614
Good	0.8 (0.4 to 1.4)	723	0.9 (0.6 to 1.3)	652
Fair	1.4 (0.7 to 2.7)	325	1.0 (0.7 to 1.6)	263
Poor	0.9 (0.4 to 2.1)	142	0.7 (0.4 to 1.3)	115
Can’t choose	1.5 (0.4 to 6.3)	14	0.3 (0.1 to 1.1)	10
Long-term limiting illness		P=0.851		P=0.09
None	1	1224	1	1117
Non-Limiting	1.1 (0.7 to 1.7)	457	1.0 (0.8 to 1.3)	401
Limiting	1.0 (0.7 to 1.7)	381	**1.4 (1.0 to 2.0)**	316
Health knowledge		P<0.001		P=0.582
Difficulty understanding information	**1.5 (1.2 to 1.9)**		1.00 (0.9 to 1.3)	
**Anxiety**				
Worry that pain is a sign of something serious		P=0.003		P=0.467
Not likely at all	1	246	1	224
Not likely	0.6 (0.3 to 1.3)	830	0.9 (0.7 to 1.3)	777
Fairly likely	1.3 (0.7 to 2.4)	622	0.9 (0.7 to 1.4)	536
Very likely	1.3 (0.7 to 2.4)	295	1.2 (0.8 to 1.8)	239
It depends	1.8 (0.8 to 4.4)	69	1.2 (0.6 to 2.2)	58
Missing				
Need to get back to normal if off work		P=0.129		P=0.14
Will not see a doctor	1	1457	1	1330
Will see a doctor if work loss	1.4 (0.9 to 2.0)	452	**1.3 (1.1 to 1.6)**	386
Will see a doctor if any to work or sleep	1.5 (0.9 to 2.5)	153	1.1 (0.7 to 1.7)	118
Missing				
**Inability to cope**				
Overwhelmed when having a health problem		P=0.002		P=0.328
SD	1	436	1	411
Disagree	1.0 (0.6 to 1.7)	797	1.0 (0.7 to 1.2)	739
Neither	**2.2 (1.3 to 3.7)**	458	1.2 (0.8 to 1.6)	379
SA/Agree	**2.0 (1.1 to 3.6)**	281	0.8 (0.6 to 1.3)	223
Never a problem	1.4 (0.6 to 3.3)	90	1.3 (0.8 to 2.2)	82
Missing				
Have someone to care for them if ill		P=0.485		P=0.27
Definitely	1	1121	1	1011
Probably	0.9 (0.6 to 1.3)	622	0.8 (0.7 to 1.1)	558
Probably not	1.2 (0.8 to 1.9)	257	0.9 (0.6 to 1.2)	217
Don’t know	1.3 (0.6 to 2.7)	62	1.3 (0.7 to 2.5)	48
Missing				
Check with family and friends for what to do		P=0.021		P=0.48
Not very likely	1	375	1	330
Not likely	0.9 (0.5 to 1.4)	552	0.8 (0.6 to 1.2)	492
Fairly likely	**0.6 (0.3 to 0.9)**	747	0.8 (0.6 to 1.1)	683
Very likely	1.1 (0.6 to 1.7)	388	0.9 (0.7 to 1.3)	327
Missing				

Factors not statistically significant in univariate analysis for either ambulance or ED: not confident in deciding when to see a doctor; did not see a doctor in the past when the health problem was serious; does not take medication to stop pain.

emboldened numbers are where 95% CI does not include 1.

SA, strongly agree; SD, strongly disagree.

In the multivariate analysis, the only patient-related factor independently associated with the tendency to attend an ED for these non-urgent scenarios was having a long-term limiting illness (OR 1.4; 95% CI 1.0 to 2.0) (table 4).

### Service-related factors associated with tendency to contact emergency services

Seven service-related factors were tested (table 5). Five of them were statistically significant in the univariate analysis for either emergency ambulance or ED. The variable about perceptions of how easy or difficult it was to get a GP appointment was not statistically significant. In the multivariable analysis, tendency to call an emergency ambulance for these non-urgent scenarios was higher for people who had been frequent users of EDs in the previous year (table 5). In the multivariable analysis, tendency to attend an ED in the study scenarios was higher for people who preferred EDs because they could get tests undertaken quickly (table 5).

**Table 5:** Service-related factors associated with tendency to contact emergency services

Factors	Ambulance versus all other options		ED versus all other options, excluding ambulance	
	Multivariable model		Multivariable model	
	OR (95% CI)	n	OR (95% CI)	n
**Preference for ED**				
Prefer ED for quick tests		P=0.115		P<0.001
Disagree/SD	1	1059	1	980
Neither	1.4 (0.9 to 2.0)	694	**1.4 (1.1 to 1.8)**	602
SA/Agree	1.5 (0.9 to 2.4)	309	**2.1 (1.5 to 2.9)**	252
Missing				
Travel to ED		P=0.297		P=0.369
Very difficult	1	418	1	351
Neither	0.7 (0.5 to 1.2)	171	0.9 (0.7 to 1.2)	149
Fairly easy	1.1 (0.7 to 1.6)	840	0.9 (0.7 to 1.2)	747
Very easy	0.9 (0.5 to 1.7)	633	0.7 (0.5 to 1.1)	587
No confidence in GP		P=0.324		P=0.425
SD	1	497	1	456
Disagree	1.2 (0.8 to 1.9)	905	0.9 (0.7 to 1.2)	806
Neither	1.0 (0.6 to 1.6)	441	0.9 (0.6 to 1.2)	383
SA/Agree	0.8 (0.4 to 1.5)	219	0.7 (0.5 to 1.1)	189
Prefer no appointments		P=0.645		P=0.499
Disagree/SD	1	612	1	563
Neither	0.9 (0.6 to 1.3)	702	0.9 (0.7 to 1.1)	630
SA/Agree	1.0 (0.7 to 1.5)	748	0.9 (0.7 to 1.2)	641
ED frequent user in the past 12 months		P=0.008		P=0.414
<3 times	1	1946	1	1742
3+ times	**2.1 (1.2 to 3.7)**	116	1.2 (0.8 to 1.9)	92

Factors not statistically significant in univariate analysis for either service: desire for services to be open at times convenient to them; ease or difficulty getting GP appointment.

emboldened numbers are where 95% CI does not include 1

SA, strongly agree; SD, strongly disagree.

### Differences by vignette

There were no statistically significant interactions between ‘vignette set’ completed and factors in the multivariable regression for ambulance services or EDs.

## Discussion

### Summary of findings

In this study presenting non-urgent healthcare scenarios to a sample of the British population, around 1 in 10 respondents selected to contact an ambulance, and 4 in 10 to attend an ED, mainly for the vignettes about fever in children and sore rib. Males, people from ethnic minority communities and older people had a higher tendency to select ambulance and ED compared with others in the population. Tendency to call an ambulance was also characterised by individuals having ‘low resources’ (manual or unskilled occupations, no car, low health literacy), distress (feeling overwhelmed by health problems) and frequent use of EDs. There appeared to be an attraction to characteristics of EDs in terms of availability of tests.

### Comparison with other research

Our population survey vignette results in Britain in 2018 were similar to a population survey vignette study of ambulance use for non-urgent problems in Japan in 2007 where respondents were more likely to select the option of calling an emergency ambulance if they were male, elderly and did not have a car.^13^ The authors of this Japanese study drew attention to the role of socioeconomic factors, estimating that these increased unnecessary ambulance use by 10%–20%. Our results also resonate with a review of ambulance use for issues that could be dealt with in primary care, where people who were anxious, lacked health knowledge and had low socioeconomic status were more likely to call an ambulance.^3^ Lack of transport has also been identified as important in another study.^14^ Health literacy has been identified as a factor in previous studies: one study found that low parental health literacy was related to unnecessary use of ambulance for children with mild acute illness.^15^ Similarly, our findings for attending EDs were similar to a previous review where a key factor was that people expected to need investigations,^4^ and similar to a population survey in Australia where people perceived care to be better in EDs.^16^

Our findings differed from other research in two ways. First, perception of timely access to general practice care at the time of the problem arising in the vignettes was not associated with tendency to use ambulances or EDs for these non-urgent vignettes. Accessibility of general practice and alternative options for help seeking is a common finding in reviews, with people complaining about their inability to obtain a timely appointment in general practice.^3–5^ In our survey reported here, we found that around half of respondents reported difficulty accessing a GP appointment.^8^ This variable may not have been associated with answers to our vignettes because people did not attempt to obtain an appointment at general practice due to the hypothetical nature of the vignettes. Second, although our finding about older people having more of a tendency to contact services was similar to another population-based vignette study for ambulance services,^13^ studies of attendance at EDs have tended to find that non-urgent contacts are characterised by younger age.^2^

Our findings from this population survey strengthen the evidence encapsulated in recent systematic reviews which identify anxiety and perceived need for investigations as factors affecting use of emergency services for non-urgent issues.^3–6^ They also highlight some issues that perhaps do not get the attention they deserve—the effect of individuals’ lack of resources, including lack of transport and lower health literacy, on tendency to use emergency ambulance services for non-urgent problems.

### Strengths and limitations

The survey was a large national sample that offered quantitative evidence of explanations of use of emergency services for minor or non-urgent problems. There were four key limitations. First, any population survey will suffer from non-response bias. The survey had a low response rate of 42% and deliberately excluded people living in institutions and homeless people because of recruitment through housing and postal sectors. Second, although patients and members of the public were involved in developing most of the questions used in the survey, they were not involved in developing the vignettes. Third, the analysis relied on hypothetical vignettes and people may behave differently in reality. Fourth, we tested 25 variables for ambulance use and 25 for ED use in the univariate analyses so we would expect around two to be statistically significant by chance when using p<0.05. This was not a problem because we then entered all variables with p<0.05 into the two multivariable analyses. If p<0.001 is applied to the final multivariable regressions then most of the variables identified would remain in those regressions.

The approach to our multivariable analysis (recommended by the statistical reviewer) yielded largely similar results to our original analysis. The main difference was that we did not include variables about people’s attitudes to whether emergency services are misused by people who do not need them. Respondents with the attitude that services are not misused had a tendency to use emergency ambulances and EDs for minor or non-urgent problems in our original multivariable analyses.

### Implications for practice or policy

A lack of personal resources appears to influence the use of emergency ambulances for minor problems, including a lack of transport and a lack of health literacy. Social and public health interventions may be necessary to address health education, public transport availability and social support in the population. In contrast, tendency to use EDs was associated with timely availability of tests. A possible solution would be to change population beliefs that tests are required for common minor problems, and thus their perception that they need to attend EDs to obtain those tests. Efforts have been made to evaluate interventions to change population attitudes, for example, a mass media campaign in Australia explaining that ambulances are for emergencies only.^17^ This identified some impact on changing attitudes towards ambulances but did not measure whether this affected demand for ambulances.

## Data Availability

Data are available in a public, open access repository. Data from all British Social Attitude Surveys are available through the UK Data Archive at https://www.data-archive.ac.uk/
